# M2 macrophage-derived exosomal miR-486-5p influences the differentiation potential of bone marrow mesenchymal stem cells and osteoporosis

**DOI:** 10.18632/aging.205031

**Published:** 2023-09-25

**Authors:** Jincheng Liu, Zhenqian Sun, Yunhao You, Lu Zhang, Dehui Hou, Guanghui Gu, Yunzhen Chen, Guangjun Jiao

**Affiliations:** 1 Department of Orthopaedics, Qilu Hospital of Shandong University, Jinan, Shandong 250012, P.R. China; 2 The First Clinical College of Cheeloo College of Medicine, Shandong University, Jinan, Shandong 250012, P.R. China

**Keywords:** exosome, miR-486-5p, BMMSCs, differentiation, osteoporosis

## Abstract

Background: An imbalance between osteogenesis and adipogenesis in bone marrow mesenchymal stem cells (BMMSCs) can cause osteoporosis. Macrophage-derived exosomes (MD-Exos) and microRNAs (miRNAs) enriched in exosomes participate in the differentiation of BMMSCs.

Methods: Bioinformatics methods were used to analyze differentially expressed miRNAs. We cocultured M2 macrophages and BMMSCs to examine the biological function of exosomal microRNA-486-5p (miR-486-5p) on BMMSCs differentiation. Gain-of-function experiments related to osteogenesis were designed to investigate the effects of exosomes carrying miR-486-5p on an ovariectomized (OVX) mice model and the direct impact of miR-486-5p on BMMSCs. A dual luciferase experiment was performed to demonstrate the target gene of miR-486-5p.

Results: Bioinformatics analysis identified high expression of miRNA-486 in M2 macrophage-derived exosomes (M2D-Exos). The *in vitro* results demonstrated that M2 macrophage-derived exosomal miR-486-5p enhanced osteogenic capacity but inhibited the adipogenesis of BMMSCs. The direct effect of miR-486-5p on BMMSCs showed the same effects. Animal experiments revealed that exosomal miR-486-5p rescued bone loss of OVX mice. SMAD2 was characterized as a target gene of miR-486-5p. Pathway analysis showed that M2 macrophage-derived exosomal miR-486-5p stimulated osteogenic differentiation via the TGF-β/SMAD2 signalling pathway.

Conclusions: Taken together, M2 macrophage-derived exosomal miR-486-5p influences the differentiation potential of BMMSCs through the miR-486-5p/SMAD2/TGF-β signalling pathway and osteoporosis.

## INTRODUCTION

The ageing of the global population continues to accelerate, increasing the prevalence of age-related chronic diseases, including osteoporosis (OP) [1]. OP is a common bone metabolic disorder characterized by reduced bone mass and degradation of bone microstructure, resulting in impaired bone strength and an increased possibility of brittle fracture [2]. Bone marrow mesenchymal stem cells (BMMSCs) easily proliferate and differentiate and can differentiate into adipose cells and osteoblasts under specific conditions [3]. Moreover, there is reciprocal inhibition between osteogenic and adipogenic differentiation. The presence of osteogenic differentiation inducers may stimulate BMMSCs towards an osteoblastic fate and vice versa [4]. The imbalance between osteogenesis and adipogenesis is the main pathogenesis of OP. Therefore, understanding the mechanisms of bone biology is essential to elucidate the pathogenesis of OP and other bone metabolism disorders and for developing new effective therapies.

Bone regeneration is affected by a variety of factors, including inflammation. Evidence has demonstrated that macrophages contribute significantly to the immunoregulation of mesenchymal stem cells and osteoblast function in skeletal remodelling and osseous repair [5]. Different phenotypes of macrophages, including the inflammatory M1 subtype and the anti-inflammatory M2 subtype, influence this regulatory process [6]. Proinflammatory macrophage responses are often associated with fracture nonunion [7], while macrophages stimulate the differentiation and activation of BMMSCs by secreting osteogenic factors and subsequently enhancing bone mineralization [8]. Studies have shown that macrophages are critical for all phases of osteogenic differentiation and promote intramembranous bone healing *in vivo* [9]. Recently, one study found that M2 macrophage-derived exosomes exert beneficial effects on osteogenesis and inhibit adipogenesis in BMMSCs [10].

Exosomes are small membrane-enclosed vesicular particles that integrate with neighbouring cells in the circulatory pathway and mediate intercellular communication [11]. Recent evidence suggests that these exosomes are effective at mediating intercell and interorgan communication by delivering vectors carrying particular microRNAs (miRNAs) [12]. Endogenously expressed miRNAs represent a small class of noncoding RNAs composed of about 22 nucleotides that repress gene expression through complementary base pairing with the target mRNAs [13]. Abnormal expression of intracellular miRNAs may alter regulatory functions at the posttranscriptional level, which may induce a series of biological functional changes and the occurrence of disease [14]. Among them, BMMSC-derived exosomal miR-29a, miR-25, and miR-206 have been shown to enhance the osteogenesis [15–17], suggesting that osteogenic differentiation is regulated by specific exosome-derived miRNAs. Evidence suggests that M2 macrophages release miRNA microvesicles that have multiple functions and play essential roles in BMMSCs differentiation [10, 18], but the specific mechanism remains unknown. The objective of this research was to investigate the molecular mechanisms of M2 macrophage-derived exosomal miRNAs in BMMSC differentiation. Our research demonstrated that microRNA-486-5p (miR-486-5p) was highly enriched in M2 macrophage-derived exosomes (M2D-Exos) and endocytosed by BMMSCs, wherein it targeted the SMAD2 gene to influence the BMMSCs differentiation.

## RESULTS

### M2 macrophage-derived exosomal miR-486-5p regulates the osteogenic and adipogenic differentiation of BMMSCs

**Figure 1:**
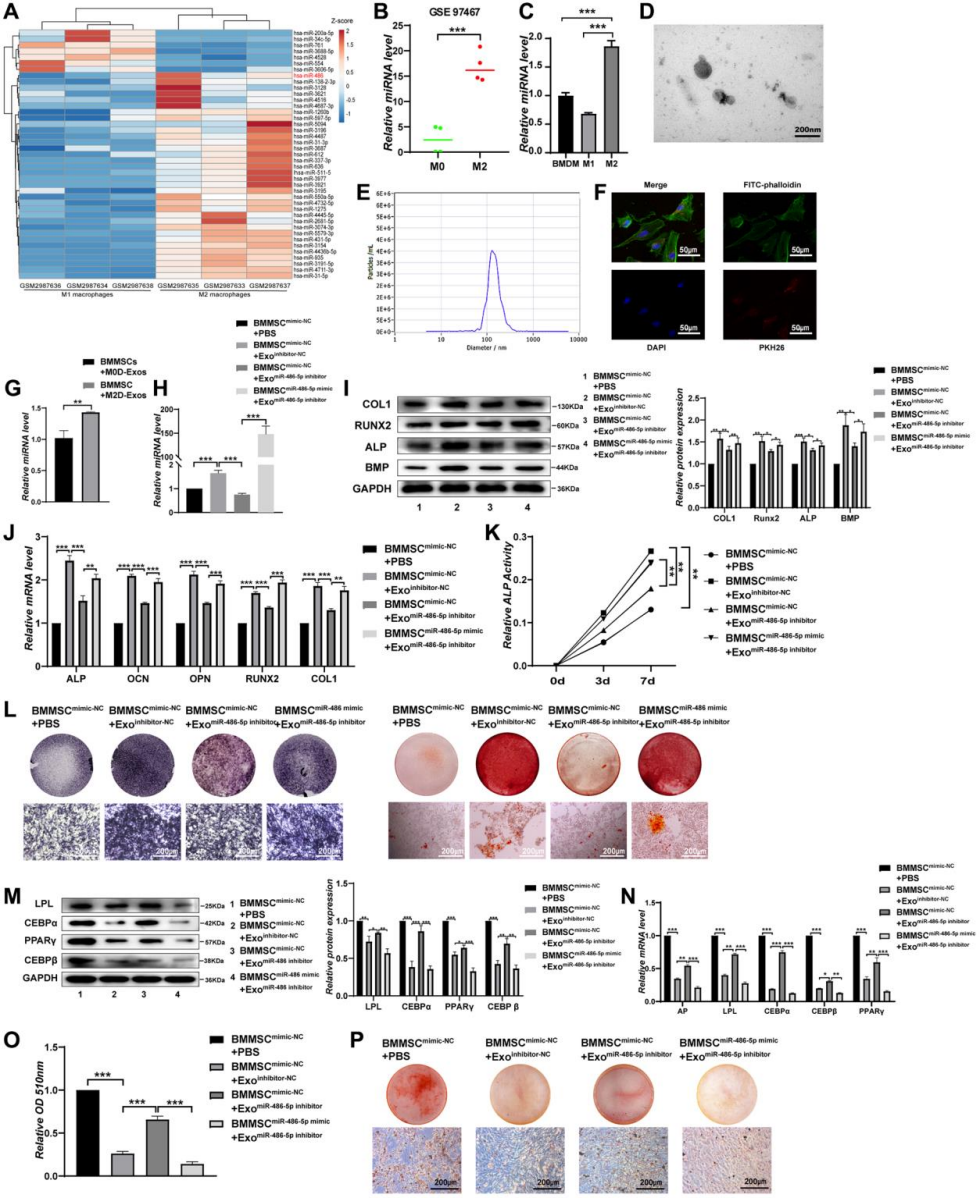
**M2 macrophage-derived exosomal miRNA-486-5p promotes the osteogenic differentiation and inhibits the adipogenic differentiation of BMMSCs.** (**A**) A heatmap identified the differently expressed miRNAs between M2 macrophages and M1 macrophages using GSE 110339 from the Gene Expression Omnibus (GEO) dataset (fold change > 1 or < − 1, Benjamini-Hochberg-corrected p). (**B**) Expression of the differentially expressed miR-486-5p between M2 macrophages-derived exosomes (M2D-Exos) and monocyte-derived exosomes using GSE97467 from the GEO dataset. (**C**) The miR-486-5p levels in bone marrow-derived macrophages (BMDMs), M1 macrophages, and M2 macrophages were measured by qRT-PCR analysis. (**D**) The morphology of M2D-Exos was shown by transmission electron microscopy (TEM). Scale bars, 200 nm. (**E**) The particle size distribution in purified M2D-Exos determined by nanoparticle tracking analysis (NTA). (**F**) Laser scanning confocal microscopy analysis of the internalization of PKH26-labelled M2D-Exos by BMMSCs, Scale bars, 50 μm. (**G**) Overexpression of miR-486-5p was detected in the BMMSCs treated with M2D-Exos by qRT-PCR analysis. (**H**) qRT-PCR analysis was used following the addition of PBS, M2D-Exos^inhibitor-NC^ (exosomes from M2 macrophages transfected with the NC inhibitor) or M2D-Exos^miR-486-5p inhibitor^ (exosomes from M2 macrophages transfected with the miR-486-5p inhibitor) to assess miR-486-5p expression in the mimic NC- or miR-486-5p-transfected BMMSCs. (**I**, **J**) The expression of osteogenic differentiation proteins and mRNAs were assessed by Western blot and qRT-PCR. (**K**) An ALP activity assay was performed to analyse ALP activity on days 0, 3, and 7. (**L**) Alizarin red staining of BMMSCs after different transfections for 21 days. Alkaline phosphatase staining of BMMSCs following different treatments for 14 days. Scale bars, 200 μm. (**M**) Western blot analysis was used to assess the expression of adipogenic differentiation proteins, including LPL, CEBPα, PPARγ, and CEBPβ. (**N**) qRT-PCR analysis of AP, LPL, CEBPα, CEBPβ, and PPARγ gene levels; (**O**, **P**) Oil red O staining and extraction were performed to detect lipid droplet formation on day 10 of adipogenic differentiation. Scale bars, 200 μm. Data are expressed as the mean ± SEM, ^*^p < 0.05, ^**^p < 0.01, ^***^p < 0.005.

We first extracted online microarray chip data from GEO (Gene Expression Omnibus, GSE110339, from https://www.ncbi.nlm.nih.gov/geo/) [19], and determined the miRNAs differentially expressed between M2 macrophages and M1 macrophages (Figure 1A). Similarly, we analysed the differentially expressed miRNAs between M2D-Exos and monocyte-derived exosomes from the Gene Expression Omnibus (GSE97467, from https://www.ncbi.nlm.nih.gov/geo/) [20]. Ultimately, the results demonstrated that miR-486 expression was mostly increased in M2 macrophages and M2D-Exos compared with their corresponding control groups (Figure 1A, 1B). We conducted subsequent experiments to evaluate miR-486 expression in bone marrow-derived macrophages (BMDMs), M1 macrophages, and M2 macrophages. As expected, miR-486 was substantially higher in M2 macrophages than in the other groups (Figure 1C). In addition, qRT-PCR demonstrated that miR-486-5p was substantially higher in M2D-Exos than in the other groups (Supplementary Figure 1A).

Subsequently, we confirmed using TEM that M2 macrophages could secrete exosomes. The TEM images revealed that most exosomes displayed a cup shape or spherical morphological shape, with an exosome diameter of about 125 nanometers (Figure 1D, 1E). The Western blot results revealed that CD63 and CD81 expression was upregulated in M2D-Exos (Supplementary Figure 1B). F4/80 and CD206 double positivity of BMDMs was markedly elevated after IL-4 treatment (Supplementary Figure 1C). This finding suggests the feasibility of the cellular M2 polarization model used in this experiment. Laser scanning confocal microscopy analysis revealed that BMMSCs efficiently internalized the M2D-Exos, as PKH26-labelled M2D-Exos were localized in the BMMSCs (Figure 1F). Next, we explored whether miR-486-5p in M2D-Exos could be successfully overexpressed in BMMSCs. qRT-PCR demonstrated that M2D-Exos increased miR-486-5p expression in BMMSCs (Figure 1G).

Exosomes were extracted from M2 macrophages transfected with a miR-486-5p inhibitor or NC inhibitor to assess the role of M2D-Exos with miR-486-5p silencing on the osteogenic differentiation of BMMSCs. qRT-PCR showed that miR-486-5p was significantly decreased by miR-486-5p knockdown in M2D-Exos, while miR-486-5p mimic transfection into BMMSCs rescued the loss of miR-486-5p in exosomes. (Figure 1H). In addition, miR-486-5p knockdown in M2D-Exos decreased the protein levels of COL1, RUNX2, ALP, and BMP, while miR-486-5p mimic transfection into BMMSCs rescued the reductions in osteogenic marker expression (Figure 1I, 1J). The mRNA expression of ALP, OCN, OPN, RUNX2, and COL1 also downregulated after miR-486-5p knockdown in M2D-Exos, while miR-486-5p mimic transfection into BMMSCs reversed this effect (Figure 1J). During osteogenic differentiation, we found that alkaline phosphatase (ALP) activity was positively related with miR-486-5p levels in M2D-Exos (Figure 1K). Alkaline phosphatase and alizarin red staining (ARS) showed that osteogenic capacity was decreased after miR-486-5p knockdown in M2D-Exos, while miR-486-5p mimic transfection into BMMSCs reversed this observation (Figure 1L).

We conducted subsequent experiments to further investigate the role of miR-486-5p in M2D-Exos on BMMSCs adipogenic differentiation. miR-486-5p knockdown in M2D-Exos increased the protein levels of LPL, CEBPα, PPARγ, and CEBPβ in BMMSCs, whereas miR-486-5p mimic transfection into BMMSCs inverted the upregulation of adipogenic marker expression (Figure 1M). qRT-PCR also suggested that miR-486-5p downregulation elevated the expression of AP, LPL, CEBPα and CEBPβ (Figure 1N). Oil red O extraction and staining revealed that miR-486-5p knockdown in M2D-Exos increased the positive rate of oil red O, while miR-486-5p mimic transfection into BMMSCs reversed this effect, indicating that M2 macrophage-derived exosomal miR-486-5p inhibited adipogenesis (Figure 1O, 1P).

### M2D-Exos-derived miR-486-5p promotes bone formation *in vivo*

**Figure 2:**
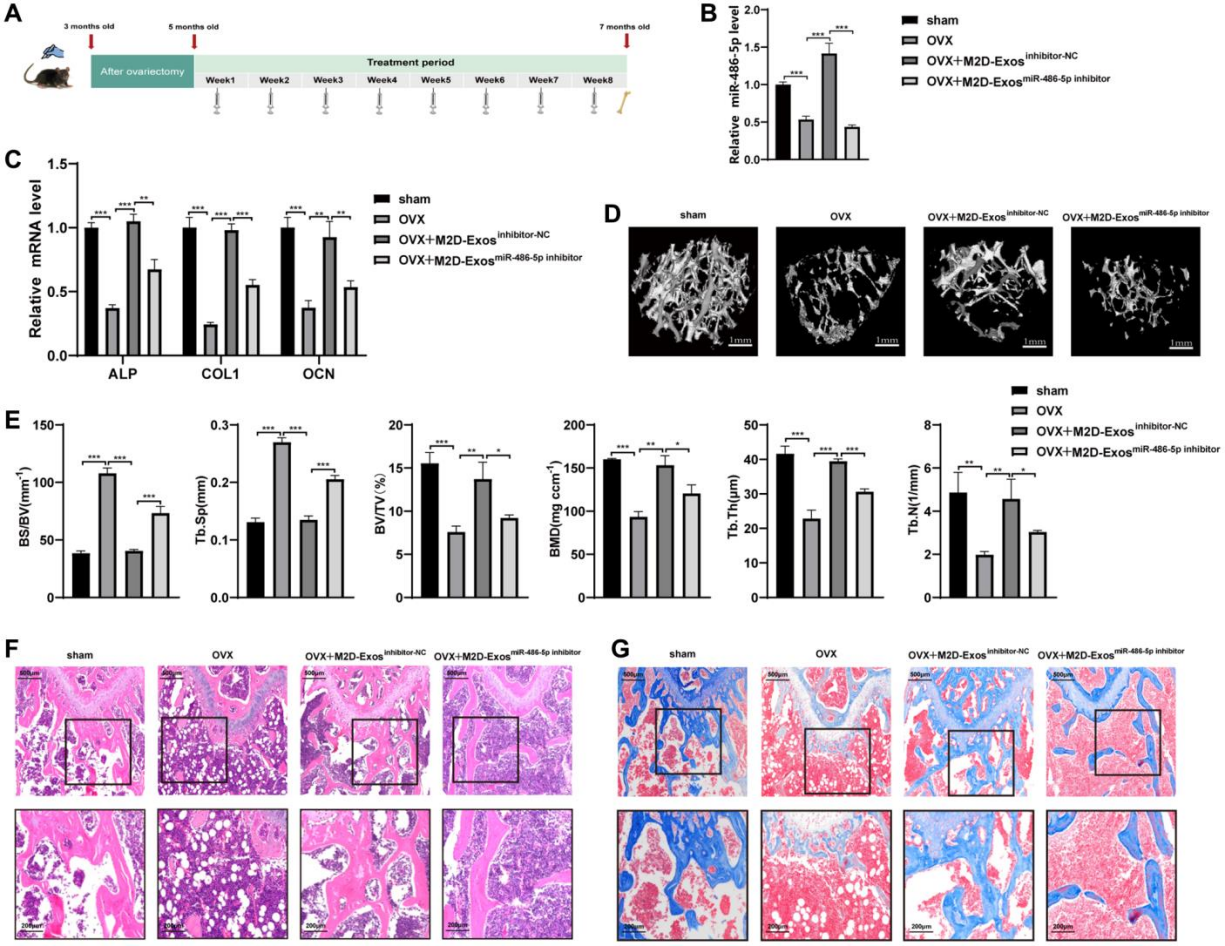
**M2D-Exo-derived miR-486-5p accelerates bone formation *in vivo*.** (**A**) Schematic diagram illustrating the experimental design. (**B**) qRT-PCR analysis of miR 486-5p levels in bone specimens from OVX mice after treatment with PBS, M2D-Exos^inhibitor-NC^, or M2D-Exos^miR-486-5p inhibitor^. (**C**) qRT-PCR analysis of ALP, OCN, and COL1 mRNA levels in bone specimens from OVX mice after treatment with PBS, M2D-Exos^inhibitor-NC^, or M2D-Exos^miR-486-5p inhibitor^. (**D**) Representative images showing the three-dimensional trabecular architecture in distal femurs determined by microCT reconstruction. Scale bars, 1 mm. (**E**) MicroCT measurements of BS/BV, Tb. Sp, BMD, BV/TV, Tb. N, and Tb. Th in the distal femurs of the OVX mice after treatment with PBS, M2D-Exos^inhibitor-NC^, or M2D-Exos^miR-486-5p inhibitor^. (**F**) H&E staining indicates trabecular density. Scale bars indicated 200 μm and 500 μm. (**G**) Masson trichrome staining indicates trabecular density and collagen. Scale bars indicated 200 μm and 500 μm. *n* = 5 mice/group. Data are expressed as the mean ± SEM, ^*^p < 0.05, ^**^p < 0.01, ^***^p < 0.005.

To further explore the therapeutic role of M2 macrophage-derived exosomal miR-486-5p on OP *in vivo*, PBS, M2D-Exos^inhibitor-NC^ (exosomes from M2 macrophages transfected with the NC inhibitor), and M2D-Exos^miR-486-5p inhibitor^ (exosomes from M2 macrophages transfected with the miR-486-5p inhibitor) were injected at equal volumes (100 μL) into three groups of 5-month-old OVX mice via their tail veins (Figure 2A). On day 14, qRT-PCR analysis was used to verify the efficiency of the miR-486-5p delivery system *in vivo*, and the results showed that miR-486-5p was increased in mice treated with M2D-Exos^inhibitor-NC^ compared with the M2D-Exos^miR-486-5p inhibitor^ group (Figure 2B). Two months postadministration, the mice treated with M2D-Exos^inhibitor-NC^ exhibited higher levels of osteogenic markers, including ALP, OCN, and COL1, while the positive effect on bone formation was mainly reversed by the downregulation of miR-486-5p in M2D-Exos (Figure 2C). We evaluated the differences in bone microstructure and microarchitectural parameters between the groups using micro-CT analysis. The results showed that the BMD, BV/TV, Tb.N and Tb.Th were substantially increased; Tb.Sp and BS/BV were notably decreased in the M2D-Exo^inhibitor-NC^ groups, and these effects were partially reversed by miR-486-5p knockdown in M2D-Exos (Figure 2D, 2E). Images with double calcein labelling also indicated that the formation of new bone was weakened after transfection with the M2D-Exo^miR-486-5P inhibitor^ (Supplementary Figure 2A). H&E and Masson's trichrome staining suggested that M2 macrophage-derived exosomal miR-486-5p facilitated bone formation in OVX mice (Figure 2F, 2G). Compared with the M2D-Exos^inhibitor-NC^ group, TRAP staining showed that the number of osteoclasts in the M2D-Exos^miR-486-5P inhibitor^ group did not change significantly (Supplementary Figure 2B). The finding suggested that the miR-486-5p in M2D-Exos may not determine the fate of bone formation by affecting the function of osteoclasts.

In addition, to elucidate the impact of miR-486-5p in M2D-Exos on adipogenic differentiation, BMMSCs were extracted from the bone marrow cavities of femurs of different groups of mice and the cells were induced towards the adipogenic differentiation. Oil red O staining revealed that miR-486-5p knockdown in M2D-Exos increased the positive rate of oil red O compared with the M2D-Exos^inhibitor-NC^ group (Supplementary Figure 2C). In addition, the adipogenic markers were increased at the protein and mRNA expression by downregulation of miR-486-5p in M2D-Exos (Supplementary Figure 2D, 2E). These results demonstrated that M2 macrophage-derived exosomal miR-486-5p had a beneficial therapeutic effect on OP *in vivo*.

### miR-486-5p induces BMMSC differentiation *in vitro*

**Figure 3:**
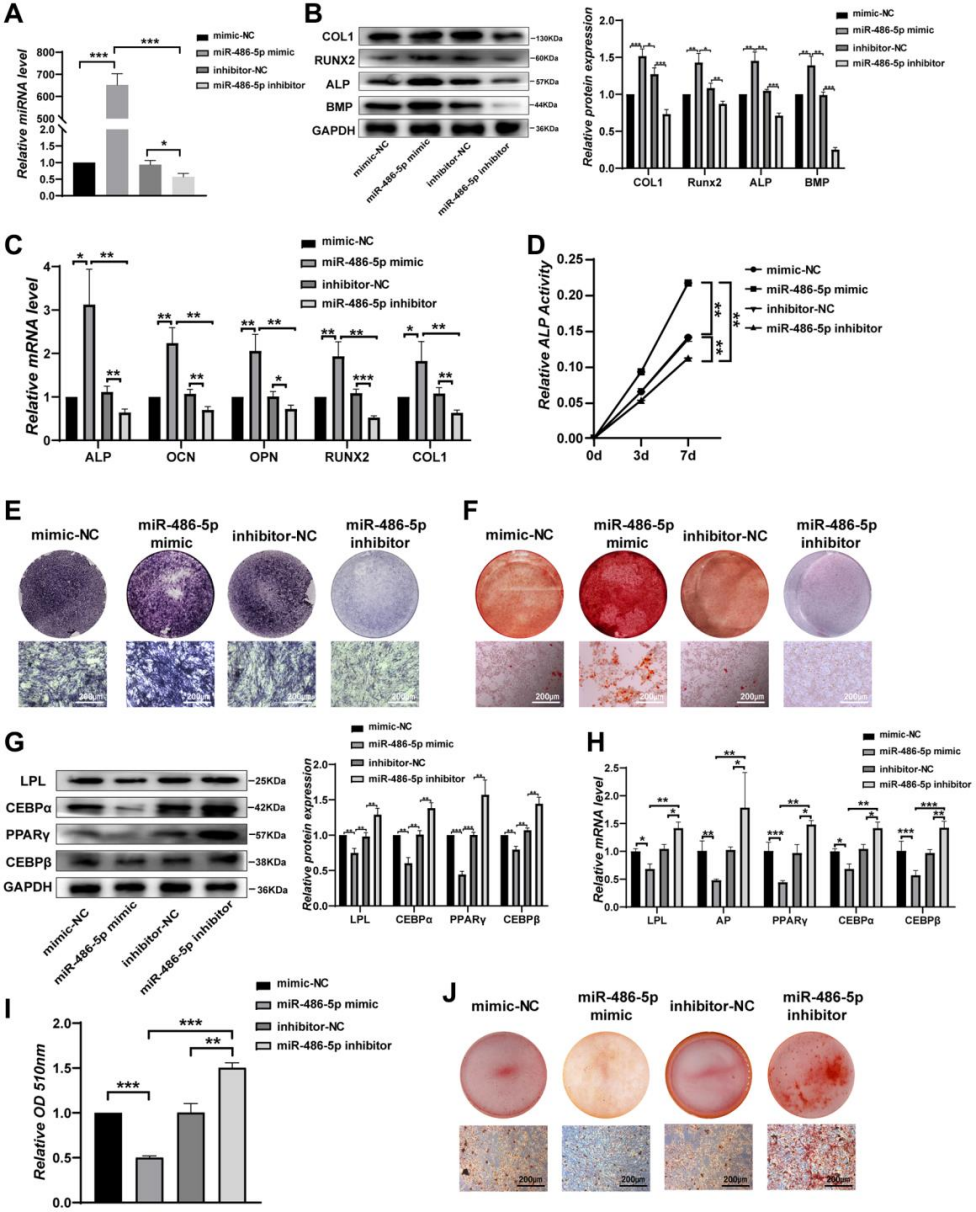
**miR-486-5p directly enhances osteogenesis but suppresses adipogenesis of BMMSCs.** (**A**) qRT-PCR analysis of miR-486-5p levels in BMMSCs after treatment with mimic-NC, miR-486-5p mimic, inhibitor-NC, or miR-486-5p inhibitor for 48 h. (**B**) Western blot analysis of COL1, RUNX2, ALP, and BMP in BMMSCs after treatment with mimic-NC, miR-486-5p mimic, inhibitor-NC, or miR-486-5p inhibitor for 48 h. (**C**) qRT-PCR analysis of the changes in the mRNA levels of the osteogenic differentiation marker genes ALP, OCN, OPN, RUNX2, and COL1 in BMMSCs after treatment with mimic-NC, miR-486-5p mimic, inhibitor-NC, or miR-486-5p inhibitor for 48 h. (**D**) Relative ALP activity was analysed during osteogenesis in treated BMMSCs on days 0, 3, and 7. (**E**) ALP staining in BMMSCs following different treatments for 14 days. Scale bars, 200 μm. (**F**) Alizarin red staining in BMMSCs after different transfections for 21 days. Scale bars, 200 μm. (**G**, **H**) The expression of adipogenic-specific markers was analysed by qRT-PCR and Western blots. (**I**, **J**) Oil red O staining and extraction were performed to detect lipid droplet formation on day 10. Scale bars, 200 μm. Data are expressed as the mean ± SEM, ^*^p < 0.05, ^**^p < 0.01, ^***^p < 0.005.

We next investigated the biological function of miR-486-5p by treating BMMSCs with NC mimics, miR-486-5p mimics, NC inhibitor, or miR-486-5p inhibitor. qRT-PCR analysis revealed that miR-486-5p was significantly upregulated by more than 600-fold after transfection with miR-486-5p mimics, while miR-486-5p levels were decreased after transfected with the miR-486-5p inhibitor (Figure 3A). BMMSCs were induced into osteogenic differentiation after transfection with miR-486-5p mimics and inhibitors. Western blot and qRT-PCR analyses showed that the levels of osteogenic transcription factors and markers were elevated after transfection with miR-486-5p mimics, whereas the opposite trend was observed after transfection with the miR-486-5p inhibitor (Figure 3B, 3C). ALP activity was positively correlated with miR-486-5p expression during osteogenic differentiation (Figure 3D). ALP staining and ARS indicated that miR-486-5p overexpression induced more pronounced ALP activity and calcium nodule formation, while miR-486-5p knockdown significantly decreased these osteogenic capabilities (Figure 3E, 3F). BMMSCs transfected with miR-486-5p mimics and inhibitors were induced to differentiate toward adipogenesis. Western blot and qRT-PCR showed that adipogenic markers was decreased by miR-486-5p upregulation and increased by miR-486-5p knockdown (Figure 3G, 3H). Similarly, oil red O extraction and staining showed that the positive rate of oil red O was reduced in miR-486-5p mimic-transfected cells and increased in miR-486-5p inhibitor-infected cells, indicating a direct suppressive role of miR-486-5p on adipogenesis in BMMSCs (Figure 3I, 3J).

### miR-486-5p directly targets SMAD2

**Figure 4:**
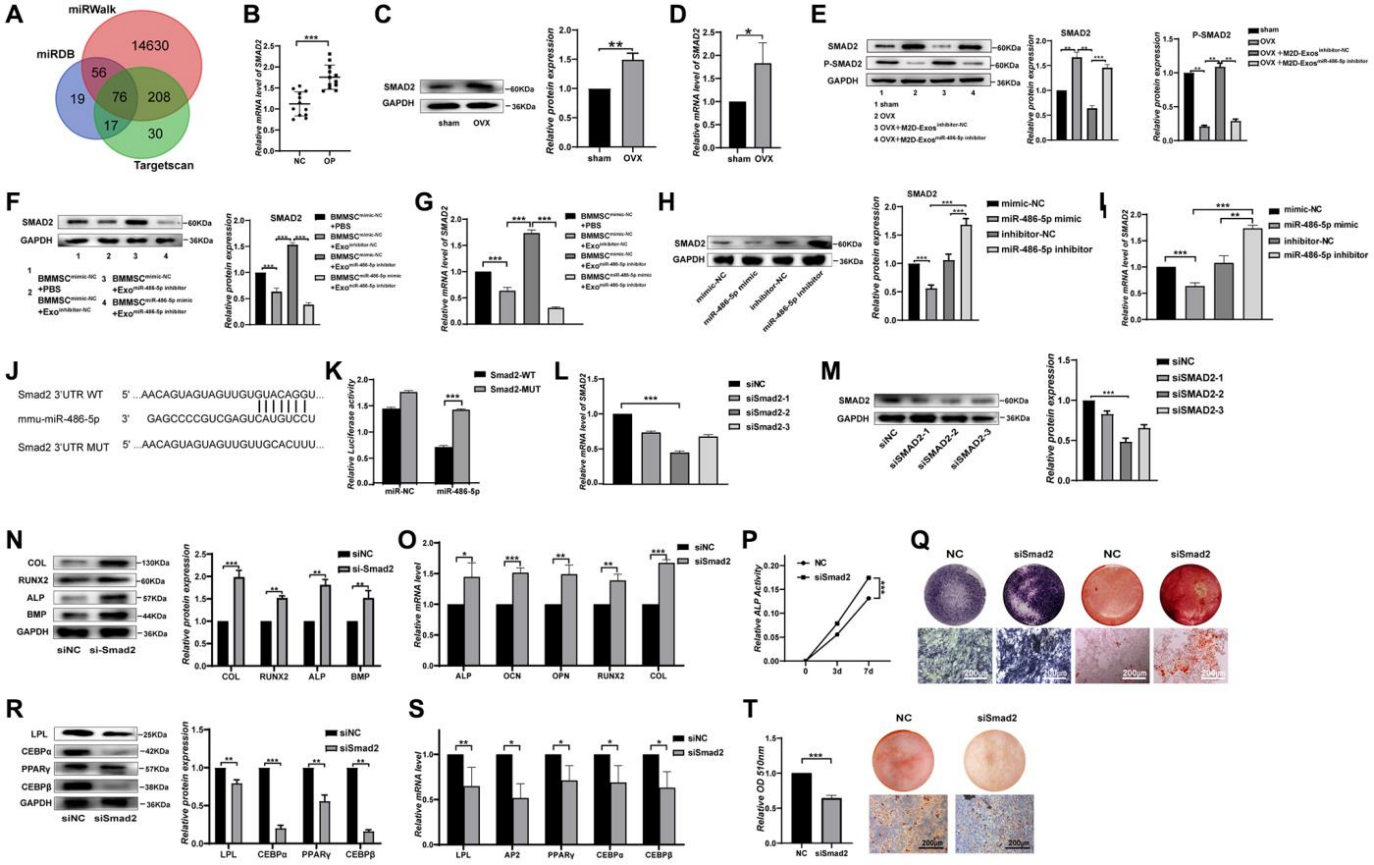
**SMAD2 was the target of miR-486-5p.** (**A**) TargetScan, miRDB, and miRWalk were used to predict gene targets of miR-486-5p. (**B**) mRNAs were extracted from bone specimens collected from female patients with osteoporosis (OP) and female subjects without osteoporosis (NC). SMAD2 mRNA expression was measured by qRT-PCR. (**C**, **D**) The expression of SMAD2 in bone tissues of OVX mice with osteoporosis and sham-operated control counterparts was measured by Western blots and qRT-PCR. (**E**) Western blot analysis was used to detect the expression of SMAD2 and p-SMAD2 in sham, OVX+PBS, OVX+M2D-Exos^inhibitor-NC^, and OVX+M2D-Exos^miR-486-5p inhibitor^ mice. (**F**, **G**) Western blots and qRT-PCR analysis were used following treatment with PBS, M2D-Exos^inhibitor-NC^, or M2D-Exos^miR-486-5p inhibitor^ to assess SMAD2 expression in mimic NC- or miR-486-5p-transfected BMMSCs. (**H**, **I**) Western blot and qRT-PCR analysis of SMAD2 levels in BMMSCs after treatment with mimic-NC, miR-486-5p mimic, inhibitor-NC, or miR-486-5p inhibitor for 48 h. (**J**) Schematic illustration of the design of luciferase reporters containing the WT SMAD2 3′UTR or the site-directed mutant SMAD2 3′UTR. (**K**) The wild-type (WT) or mutant-type (MUT) constructs were inserted into the psiCHECK-2 reporter vector. Luciferase activity was measured in the lysates, and the values were normalized to that of the psiCHECK-2 vector. (**L**, **M**) The knockdown efficiency of three SMAD2 siRNAs was confirmed by qRT-PCR and Western blot analysis. (**N**, **O**) Western blots and qRT-PCR were used to analyse osteogenic-specific markers after SMAD2 knockdown. (**P**) An ALP activity assay was performed to analyse ALP activity on days 0, 3, and 7. (**Q**) ALP staining was performed on day 14, and Alizarin red staining showed increased calcification on day 21 after SMAD2 knockdown. Scale bars, 200 μm. (**R**, **S**) Western blots and qRT-PCR were used to analyse adipogenic-specific markers after SMAD2 knockdown. (**T**) Oil red O staining and extraction were performed to detect the formation of lipid droplets on day 10 of adipogenic differentiation. Scale bars, 200 μm. Data are expressed as the mean ± SEM, ^*^p < 0.05, ^**^p < 0.01, ^***^p < 0.005.

To further clarify the specific mechanism of miR-486-5p in BMMSCs differentiation, we used TargetScan [21] (http://www.targetscan.org), miRDB [22] (http://www.targetscan.org), and miRWalk (23, 24) [23, 24] (http://mirwalk.umm.uni-heidelberg.de) to determine the potential targets of miR-486-5p, yielding 76 predicted target mRNAs (Figure 4A). A literature review revealed that SMAD2 is the most relevant candidate gene for osteogenic differentiation [25]. SMAD2 expression in OP bone tissue was higher than that in control group, implying that SMAD2 might play a significant role in osteoporosis (Figure 4B–4D). Compared with the M2D-Exos^inhibitor-NC^ group, SMAD2 expression was increased and phosphorylated SMAD2 (p-SMAD2) expression was reduced after treatment with the M2D-Exos^miR-486-5p inhibitor^ *in vivo* (Figure 4E). Western blot and qRT-PCR analyses revealed that miR-486-5p knockdown in M2D-Exos increased SMAD2 expression in BMMSCs, while miR-486-5p mimic transfection into BMMSCs reversed SMAD2 upregulation, suggesting that miR-486-5p might repress SMAD2 expression (Figure 4F, 4G). Moreover, we found that SMAD2 level was upregulated by miR-486-5p knockdown and reduced by miR-486-5p overexpression in BMMSCs (Figure 4H, 4I). SMAD2 has a miR-486-5p binding site in its 3′ untranslated region (UTR). Thus, to determine whether miR-486-5p directly targets SMAD2, dual-luciferase reporter genes with miR-486-5p binding sites in wild-type (WT) 3′UTR and mutant 3′UTR (MUT) sequences were constructed (Figure 4J). The dual-luciferase assay results revealed that miR-486-5p up-regulation attenuated the luciferase reporter activity of the WT SMAD2 3′UTR reporter but not that of the MUT SMAD2 3′UTR reporter (Figure 4K). In contrast, the NC mimics did not affect luciferase activity after cotransfection with the WT or MUT SMAD2 3′UTR. siSMAD2-2 had the most significant inhibitory effect at the mRNA and protein levels (Figure 4L, 4M), and siSMAD2-2 was therefore selected for subsequent functional experiments. SMAD2 knockdown increased the osteogenic markers at the protein and mRNA levels in BMMSCs (Figure 4N, 4O). In addition, we found that ALP activity was increased after SMAD2 knockdown during osteogenic differentiation (Figure 4P). ALP staining and ARS staining also showed that osteogenic capacity was significantly enhanced in BMMSCs treated with siSMAD2-2 (Figure 4Q). To assess the role of SMAD2 on the adipogenic differentiation, BMMSCs were induced to differentiate into adipocytes after SMAD2 knockdown. Depletion of SMAD2 significantly decreased adipogenic markers at the protein and mRNA levels (Figure 4R, 4S). Oil red O extraction and staining revealed that SMAD2 knockdown significantly decreased the positive rate of oil red O (Figure 4T). In conclusion, these results suggest that SMAD2 knockdown markedly decreased the adipogenic differentiation of BMMSCs.

### SMAD2 knockdown reverses the differentiation induced by miR-486-5p in BMMSCs

**Figure 5:**
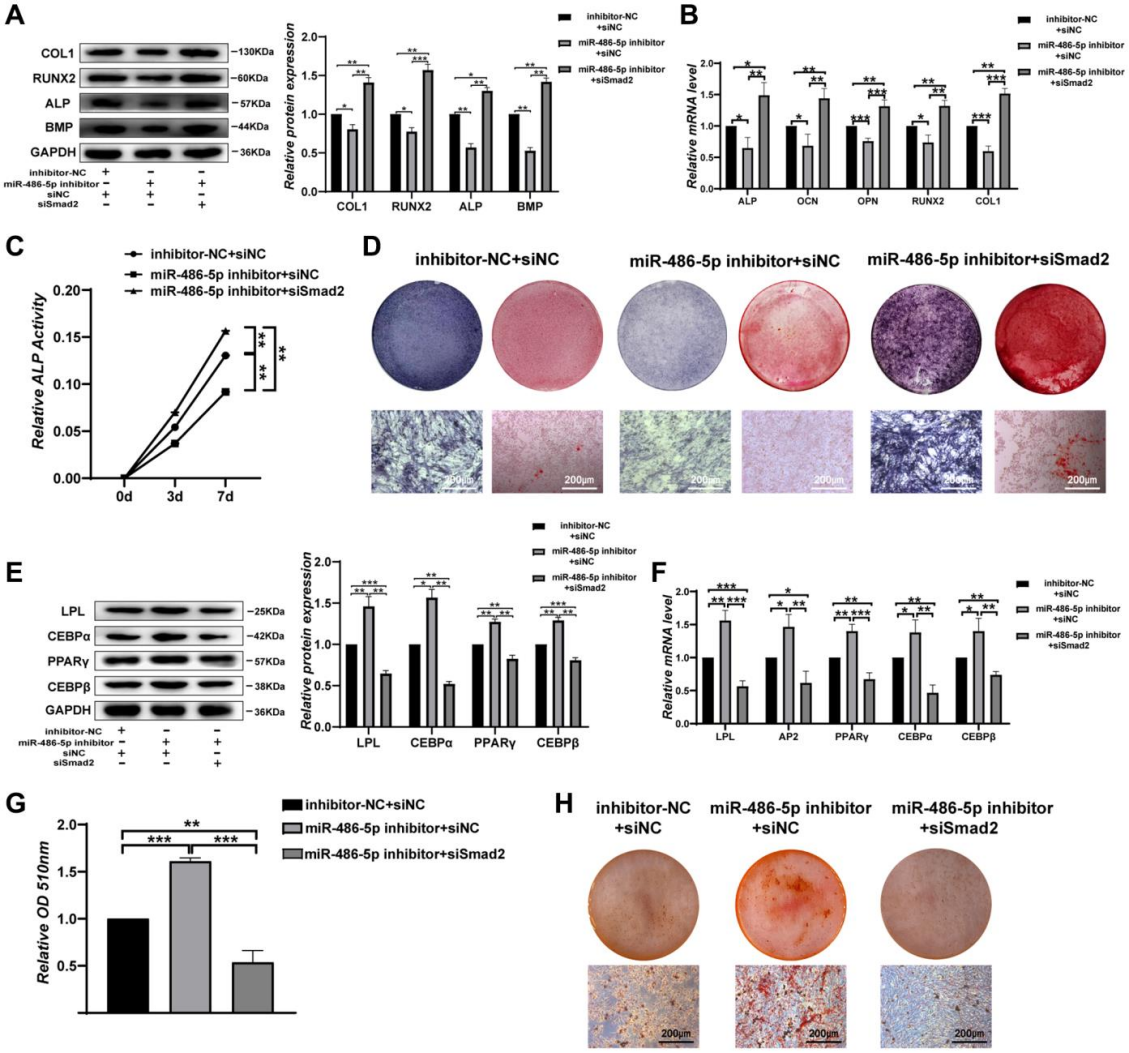
**SMAD2 knockdown reverses the effect of downregulated miR-486-5p expression on BMMSC differentiation.** (**A**, **B**) Western blots and qRT-PCR were used to analyse osteogenic factor expression after different treatments. (**C**) ALP activity assays were performed to analyse ALP activity during osteogenic differentiation on days 0, 3, and 7. (**D**) ALP staining was performed on day 14, and Alizarin red staining showed increased calcification on day 21 after different transfections. Scale bars, 200 μm. (**E**, **F**) Western blotting and qRT-PCR were performed to analyse the protein and mRNA expression levels of adipogenic markers, respectively. (**G**, **H**) Oil red O staining and extraction were performed to detect lipid droplet formation on day 10 of adipogenic differentiation. Scale bars, 200 μm. Data are expressed as the mean ± SEM. ^*^p < 0.05, ^**^p < 0.01, ^***^p < 0.005.

We next investigate the impact of SMAD2 on BMMSCs differentiation. When siSMAD2-2 was transfected into miR-486-5p knockdown cells, we found that SMAD2 downregulation partly reversed the osteogenic capacity decreased by miR-486-5p knockdown, as indicated by the upregulated osteogenic-related genes at the protein and mRNA levels (Figure 5A, 5B). ALP activity reduction due to miR-486-5p knockdown was rescued by SMAD2 knockdown during osteogenic differentiation (Figure 5C). Moreover, ALP staining and calcium nodule formation were enhanced by SMAD2 knockdown (Figure 5D). In addition, SMAD2 knockdown reversed the promotion of adipogenic abilities induced by miR-486-5p knockdown, mainly manifested as the downregulated the adipogenesis-specific factors at the mRNA and protein levels (Figure 5E, 5F). Oil red O extraction and staining showed that SMAD2 knockdown increased the positive rate of oil red O (Figure 5G, 5H). Hence, these data confirmed that SMAD2 deletion abolished the effect of miR-486-5p downregulation.

### miR-486-5p regulates the osteogenic and adipogenic differentiation of BMMSCs through the TGF-β signalling pathway

**Figure 6:**
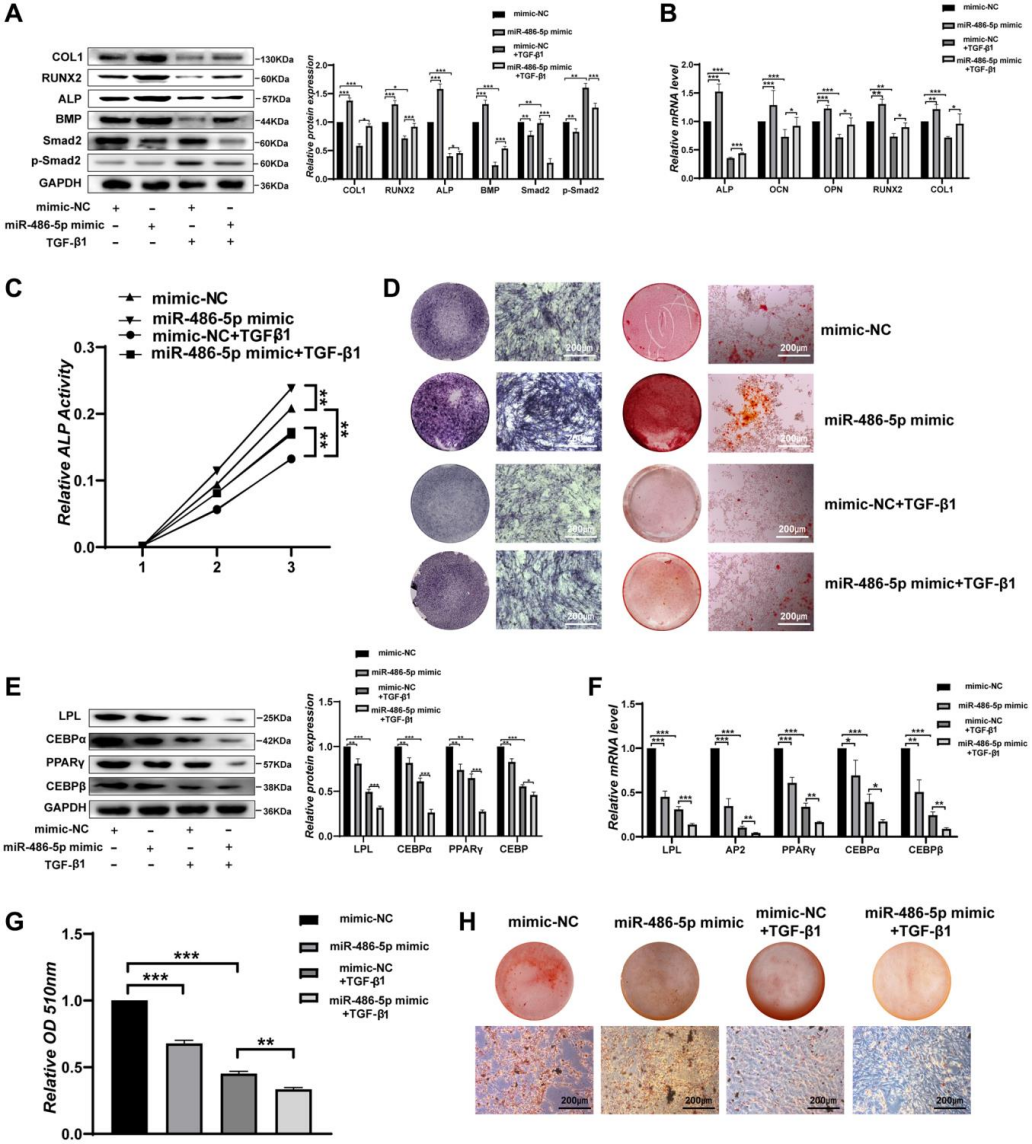
**miR-486-5p regulates BMMSC differentiation through the TGF-β signalling pathway.** (**A**) Western blot analysis of the protein levels of osteogenic and TGF-β signalling pathway-related molecular elements after different treatments. (**B**) The mRNA levels of osteogenic genes after different treatments. (**C**) ALP activity assays were performed to analyse ALP activity in the treated BMMSCs on days 0, 3, and 7. (**D**) ALP staining was performed on day 14, and Alizarin red staining showed increased calcification on day 21 after different treatments. Scale bars, 200 μm. (**E**, **F**) Western blotting and qRT-PCR were performed to analyse the expression levels of adipogenic factors after different treatments. (**G**, **H**) Oil red O staining and extraction were performed on day 10 of adipogenic differentiation after different treatments. Scale bars, 200 μm. Data are expressed as the mean ± SEM. ^*^p < 0.05, ^**^p < 0.01, ^***^p < 0.005.

SMAD2 is considered an important modulator of the transforming growth factor β (TGF-β) signalling pathway, and p-SMAD2 acts as a functional factor in the TGF-β signalling pathway that modulates the osteogenic differentiation of BMMSCs. To further investigate whether miR-486-5p affects BMMSCs differentiation through the TGF-β signalling pathway, BMMSCs were stimulated by TGF-β1. Consistent with previous studies [26–28], we found that a high concentration (5–15 ng/mL) of TGF-β1 inhibited the osteogenesis of BMMSCs (Supplementary Figure 3A–3C). The protein expression of osteogenic markers was downregulated and p-SMAD2 expression increased after transfection with TGF-β1 in BMMSCs, while miR-486-5p overexpression rescued these osteogenic capabilities (Figure 6A). qRT-PCR also indicated that miR-486-5p upregulation significantly increased the osteogenic capabilities decreased by TGF-β1 (Figure 6B). ALP activity reduction due to TGF-β1 was rescued by miR-486-5p overexpression during osteogenic differentiation (Figure 6C). ALP expression and calcium nodule formation were decreased after transfection with TGF-β1 in BMMSCs, while miR-486-5p overexpression reversed the effects of TGF-β1 on osteogenic differentiation (Figure 6D). However, the decreased adipogenic differentiation induced by TGF-β1 was further promoted by miR-486-5p overexpression, as evidenced by decreased adipogenic marker expression and a substantial reduction in the number of oil red O-positive adipocytes (Figure 6E–6H). These data demonstrated that the negative effects of osteogenic differentiation by TGF-β1 were almost blocked by miR-486-5p overexpression, while the roles of TGF-β1 on adipogenic differentiation were not markedly weakened by miR-486-5p overexpression. These findings further suggested that miR-486-5p promotes osteogenic differentiation through the TGF-β signalling pathway, while the inhibition of adipogenic differentiation may be mediated primarily by other pathways.

## DISCUSSION

OP has become a growing public health problem as the population ages [29]. OP is characterized by the degradation of bone microstructure and excessive accumulation of adipocytes in the bone marrow cavity, leading to an increased risk of fragility fractures in older patients [30, 31]. OP occurs most frequently in postmenopausal women and elderly men [32]. The increased ability of BMMSCs to differentiate into adipocytes and the decreased ability to differentiate toward osteoblasts is an important mechanism leading to bone loss in OP [33]. In this study, we found a new mechanism whereby miR-486-5p in M2D-Exos participated in this change in the cell lineage commitment of BMMSCs. Furthermore, M2D-Exos served as a BMMSC-specific delivery system to deliver miR-486-5p to OVX mice.

The changing polarization state of macrophages rapidly senses and responds to local variations to maintain microenvironmental homeostasis [8]. Macrophages mainly have two polarization states, including M1 macrophages and M2 macrophages. Modulation of macrophage polarity to ameliorate the inflammatory milieu is an effective therapeutic approach to disease. M1 macrophages are associated with pro-inflammatory responses, while M2 macrophages are primarily involved in anti-inflammatory responses [34]. Mahon OR et al. found that M2 macrophages have a positive regulatory role in osteoporosis [35–37]. Recently, Li Z et al. observed that exosomes derived from M2 macrophages promoted osteogenesis and decreased adipogenesis in BMMSCs [10]. As important paracrine factors, these small vesicles can modulate the function of receptor cells during internalization and thus mediate intercellular communication in the bone microenvironments [38, 39]. In this study, we cocultured macrophages and BMMSCs, demonstrating that exosomes from M2 macrophages could be endocytosed by BMMSCs and then regulate BMMSCs differentiation.

miRNAs are essential for gene expression and have been shown to be involved in metabolic disease including OP [14]. Evidence has also showed that miRNAs in cell-released exosomes can be delivered into acceptor cells and subsequently modulate neighbouring cells and distant cells [39]. In our research, miR-486-5p was considered as a key miRNA overexpressed in M2D-Exos that regulates BMMSCs differentiation. Previously, miR-486-5p in stem cell-derived exosomes was shown to regulate angiogenesis of endothelial cells and suppress the MMP19 transition [40]. However, there is no report on the effect of the M2 macrophage-derived exosomal miR-486-5p in regulating BMMSC function. In this study, we defined a new mechanism whereby M2 macrophage-derived exosomal miR-486-5p promotes osteogenesis and suppresses adipogenesis in BMMSCs. We further observed that the M2 macrophage-derived exosome miR-486-5p reduced bone loss in OVX mice.

miRNAs are non-coding RNAs with regulatory functions that repress gene expression through complementary base pairing with target mRNAs [41]. Therefore, we used TargetScan, miRDB, and miRWalk to determine the sites of miR-486-5p and found that the SMAD2 was a target gene of miR-486-5p. SMAD2, which shows dramatically downregulated expression during osteogenesis, inactivates RUNX2 to repress osteogenesis and promote the adipogenesis of BMMSCs, which may be achieved via the recruitment of class II HDACs 4 and 5 by activated SMAD2/3 to repress RUNX2 activity [42]. Our study suggested that SMAD2 levels were downregulated in OP bone samples. Luciferase reporter assays also showed that the 3′UTR of SMAD2 mRNA could be bound by miR-486-5p. In addition, we also provided proof that miR-486-5p enhanced osteogenesis and suppressed adipogenesis by targeting SMAD2 in BMMSCs.

Smad family proteins are involved in TGF-β signalling, including the transmission of signals from cell surface receptors to the cell nucleus [43]. TGF-β is a ligand for the formation of receptor complexes that activate Smads in the nucleus and coactivate or suppress the transcription of the target genes they regulate [44]. We observed a higher abundance of p-SMAD2 in the TGF-β1-treated cells, and p-SMAD2 repressed RUNX2 expression during the osteogenic differentiation of BMMSCs. TGF-β1 is the most abundant isomer, and bone (200 g/kg) is one of the major sources of TGF-β1 [45, 46]. Osteoblasts and BMMSCs secrete TGF-β1, and deposit it in the bone matrix. TGF-β signaling is essential for the differentiation of BMMSCs into osteoblasts *in vivo*, and TGF-β acts efficiently on osteoblasts to modulate the remodelling, microarchitecture, and mechanical properties [47]. Previous studies have found that a high concentration (5–25 ng/mL) of TGF-β1 inhibits osteogenic differentiation [26–28]. In our experiments, 5 ng/mL TGF-β1 was used to stimulate BMMSCs. We found that high TGF-β1 concentrations strongly inhibited the osteogenesis and adipogenesis of BMMSCs and that the upregulation of p-SMAD2 expression decreased miR-486-5p overexpression-mediated promotion of osteogenesis, further suggesting that miR-486-5p regulates osteogenic differentiation via the SMAD2/TGF-β signaling pathway. However, the suppression of adipogenesis by miR-486-5p overexpression was not substantially weakened by upregulation of p-SMAD2 expression. This finding suggested that another pathway may participate in regulating the adipogenic differentiation of BMMSCs.

## CONCLUSIONS

**Figure 7:**
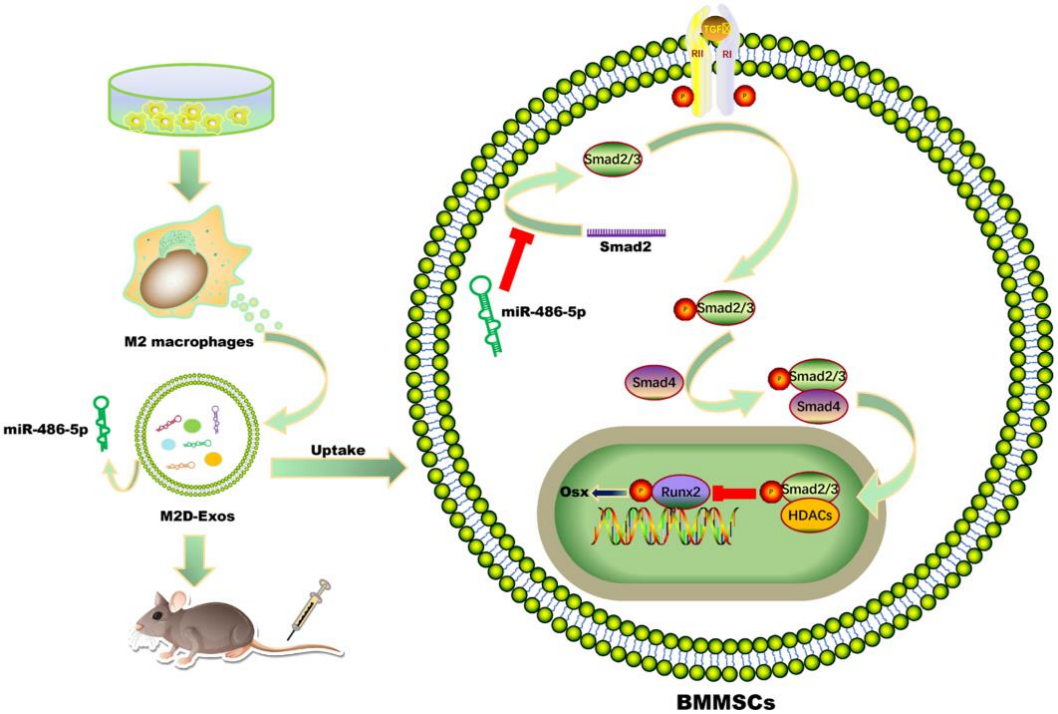
**Schematic diagram showing the proposed mechanisms by which exosomal miR-486-5p derived from M2 macrophages regulates the osteogenic and adipogenic differentiation of BMMSCs.** miR-486-5p enrichment in M2D-Exos enhances the inhibitory effect on SMAD2, resulting in downregulation of the TGF-β signalling pathway and thereby accelerating bone remodelling.

In summary, these findings indicated that exosomal miR-486-5p in M2 macrophage modulates the BMMSCs differentiation by the SMAD2/TGF-β signaling pathway (Figure 7). This exosomal miR-486-5p exerts a critical role in OP by promoting the osteogenic differentiation of BMMSCs, and may represent a new feasible treatment strategy for osteoporosis.

## MATERIALS AND METHODS

### Cell culture and treatment

We used 3-week-old C57BL/6J mice for cell extraction and obtained approval from the Animal Ethics Committee of Qilu Hospital, Shandong University (DWLL-2021-136). After administering Pelltobarbitalum Natricum (50 mg/kg; Sigma-Aldrich, St. Louis, MO, USA) to euthanize the mice, we carefully isolated the femur and flushed the bone marrow cavity using the basal medium. We used RPMI-1640 and DMEM/F12 (1:1) medium to culture BMDMs and BMMSCs respectively. 10% Fetal Bovine Serum (FBS), and 1% antibiotics were added to the basal medium. Bone marrow cells were cultured for 7 days to obtain BMDMs by adding 50 ng/mL M-CSF (#216-MC, R&D System, Minneapolis, MN, USA) to RPMI-1640 as previously described [18]. Subsequently, 20 ng/ml IL4 was added and incubated for 24 hours to induce differentiation of BMDMs to M2 macrophages. We examined M2 macrophage differentiation using flow cytometry. Place the cells in the incubator and set the temperature to 37°C. The medium was changed regularly and passaged after digestion using trypsin (#25200056, Gibco, Rockville, MD, USA). Cells within 5 generations were selected for the experiment.

### Isolation and identification of exosomes

M2 macrophages were cultured for 72 h to 80% confluence and then centrifuged at 300 g for 10 min. We removed dead cells from the supernatant by centrifugation at 16,500 g and 4°C and then filtered the supernatant using a 0.22-μm filter. The samples were then ultracentrifuged at 100,000 g for 70 min at 4°C to collect the precipitate. Ultimately, three different exosome populations were obtained: M2D-Exos, M2D-Exos^inhibitor-NC^ (exosomes from M2 macrophages transfected with the NC inhibitor), and M2D-Exos^miR-486-5p inhibitor^ (exosomes from M2 macrophages transfected with the miR-486-5p inhibitor). Transmission electron microscopy (TEM; EFI, TECNAI G2, USA) and nanoparticle tracking analysis (NTA; ZetaVIEW, Germany) were used to examine exosome morphology.

### M2 macrophage exosome uptake by BMMSCs

The isolated exosomes were labelled with PKH 26 (#MINI26-1KT, Sigma–Aldrich, St. Louis, MO, USA) and used to treat BMMSCs for 12 h. The treated cells were fixed with 4% paraformaldehyde and then immersed with 0.5% Triton-X 100 (#T8200, Solarbio, China). Cells were subsequently treated with phalloidin (#CA1620, Solarbio, China) and DAPI (#C0065, Solarbio, China) and visualized using confocal microscopy (Zeiss, LSM780, Germany).

### microRNA mimics, inhibitors, and siRNA transfection

We used Lipofectamine 2000 (#11668030, Invitrogen, Carlsbad, CA, USA) to transfect microRNA mimics, inhibitors, and siRNAs into the cells. Added miR-486-5p mimics, miR-486-5p inhibitor, SMAD2 siRNA, and negative controls (GenePharma, China) at a concentration of 20 μM to the medium to transfect into BMMSCs. M2 macrophages were treated with miR-486-5p inhibitor or NC inhibitor. BMMSCs transfected with the NC mimics (BMMSCs^mimics-NC^) were treated with PBS, M2D-Exos^inhibitor-NC^, or M2D-Exos^miR-486-5p inhibitor^. BMMSCs transfected with miR-486-5p mimics (BMMSCs^miR-486-5p mimics^) were treated with an M2D-Exos^miR-486-5p inhibitor^.

### Western blot analysis

1% PMSF (#ST506, Beyotime, China) was added to the RIPA lysate for lysing cells and tissues. The lysate was then centrifuged at 12,000 g to obtain total protein. Western blotting experiments was carried out based on previous studies [18]. The antibodies Smad2(#5339, 1:1000), RUNX2(#12556, 1:1000), CEBP-α (#8178S, 1:1000), CEBP-β (#43095S, 1:1000), PPARγ (#2443S, 1:1000) and Phospho-Smad2 (#3108S, 1:1000) were purchased from Cell Signaling Technology (Danvers, MA, USA). The antibodies BMP-2 (#ab284387, 1:1000) and LPL (#ab91606, 1:1000) were purchased from Abcam (Cambridge, UK). The antibody ALP (#DF6225, 1:1000) was purchased from Affinity Bioscience (Jiangsu, China). The antibody GAPDH (#bsm-33033M, 1:5000) was purchased from Bioss (Beijing, China). After incubation of the secondary antibody, visualisation was performed using ChemiDoc Touch Gel Imaging System (Tanon, Shanghai, China).

### RNA extraction and qRT-PCR

The TRIzol (#15596026, Invitrogen, Carlsbad, CA, USA) was used to extract RNA from cells and bone tissue and determined RNA content using spectrophotometer. ReverTrace qPCR RT Kit (#TRT-101, TOYOBO, Japan) was performed to reverse transcribe to complementary DNA. We performed qRT-PCRs using the corresponding primers in a LightCycler 480 system (Roche). The sequences of the primers are shown in Table 1.

**Table 1:** The primer sequence of the study.

**Name**	**Sequence**
ALP Primer	F: GCACCTGCCTTACCAACTCT
	R: GTGGAGACGCCCATACCATC
OCN Primer	F: TCTGACCTCACAGATGCCAAG
	R: AGGGTTAAGCTCACACTGCT
OPN Primer	F: CACATGAAGAGCGGTGAGTCT
	R: CCCTTTCCGTTGTTGTCCTG
RUNX2 Primer	F: GGGACTGTGGTTACCGTCAT
	R: ATAACAGCGGAGGCATTTCG
COL1 Primer	F: CCCTGGTCCCTCTGGAAATG
	R: GGACCTTTGCCCCCTTCTTT
GAPDH Primer	F: GGTCACCAGGGCTGCTTTTA
	R: GGATCTCGCTCCTGGAAGATG
LPL Primer	F: ACAAGAGAGAACCAGACTCCAA
	R: AGGGTAGTTAAACTCCTCCTCC
AP2 Primer	F: AGCACCATAACCTTAGATGGGG
	R: CGTGGAAGTGACGCCTTTCA
PPARγ Primer	F: GCCGAGTCTGTGGGGATAAA
	R: TCCGGCAGTTAAGATCACACC
CEBPα Primer	F: AGGAACACGAAGCACGATCAG
	R: CGCACATTCACATTGCACAA
CEBPβ Primer	F: CTTCAGCCCGTACCTGGAG
	R: GGAGAGGAAGTCGTGGTGC
Smad2 Primer	F: CGTCCATCTTGCCATTCACG
	R: CTCAAGCTCATCTAATCGTCCTG
miR-486-5p Primer	F: ACATGCAATTTCCTGTACTGAGC
	R: TATGGTTGTTCTCGTCTCTGTGTC
miR-486-5p mimic	UCCUGUACUGAGCUGCCCCGAG
	CGGGGCAGCUCAGUACAGGAUU
miR-486-5p inhibitor	CUCGGGGCAGCUCAGUACAGGA
m-U6 Primer	F: CAGCACATATACTAAAATTGGAACG
	R: ACGAATTTGCGTGTCATCC
siSmad2-1	F: CCAAGCACUUGCUCUGAAATT
	R: UUUCAGAGCAAGUGCUUGGTT
siSmad2-2	F: GGUGUUCGAUAGCAUAUUATT
	R: UAAUAUGCUAUCGAACACCTT
siSmad2-3	F: CCCUGCAACAGUGUGUAAATT
	R: UUUACACACUGUUGCAGGGTT

Abbreviations: F: forward; R: reverse; ALP: Alkaline Phosphatase; OCN: osteocalcin; OPN: osteopontin; Runx2: runt-related transcription factor 2; COL: collagen; LPL: lipoprotein lipase; AP2, namely FABP4: fatty acid binding protein 4; PPARγ: peroxisome proliferator-activated receptor gamma; CEBPα/β: CCAAT/enhancer binding protein α/β.

### Induction of BMMSC differentiation

Dexamethasone (10 nM), ascorbic acid (0.25 mM) and sodium β-glycerophosphate (10 mM) were added to DMEM/F12 complete medium to form an osteogenic induction solution. To induce adipogenic differentiation of BMMSCs, we added 3-Isobutyl-1-methylxanthine (0.5 mM), insulin (10 μg/mL), dexamethasone (1 μM), and indomethacin (0.2 mM) to the complete medium to culturing cells. After performing 10 consecutive days of culture, we performed Oil Red O staining to assess adipogenic differentiation.

### Alizarin red staining and alkaline phosphatase staining

BMMSCs were treated with osteogenic differentiation induction medium. After 21 days, the cells were fixed with 4% formaldehyde for 30 min and washed 3 times with PBS. Alizarin red was used to stain treated cell for 30 min. After 14 days of BMMSCs induction, cells from different groups were treated with alkaline phosphatase chromogenic kit (#C3206, Beyotime, China). The images of stained cells were taken with a microscope (ZEISS, Axio Vert.A1, Germany).

### Oil red O staining

BMMSCs were treated with adipogenic differentiation induction medium. After 10 days, the treated cells were fixed with 4% paraformaldehyde for 30 min at room temperature, rinsed with water and stained with an oil red O staining (#G1262, Solarbio, China). After washing with deionized water, oil red O-positive cells were visualized using a microscope (ZEISS, Axio Vert.A1, Germany).

### Alkaline phosphatase assay

After discarding the culture medium, BMMSCs were soaked in 1% Triton X-100. Quantitative ALP activity was measured using commercial kits (#P0321M, Beyotime Institute of Biotechnology, China), and the absorbance was measured at 405 nm using a spectrophotometer (Bio-Rad, Hercules, CA, USA).

### Mice model of osteoporosis

The mice experiments were approved by the Animal Ethics Committee of Qilu Hospital of Shandong University (DWLL-2021-136). In addition, we complied with the ARRIVE guidelines for animal experiment. Fifty female C57BL/6 mice (three months old) were randomly divided into two groups: the sham-operated group (*n* = 15) and the ovariectomized (OVX) group (*n* = 35). All mice undergoing surgical manipulation were anesthetized using sodium pentobarbital (50 mg/kg). A 20-mm incision was made in the lumbar dorsum of the mice, and the ovaries were located on the fat pads at the lower edge of the kidneys. In the ovariectomy group, we ligated the blood vessels and oviducts and then resected both ovaries. In the group of sham-operated mice, we cut off part of the fat pad near the ovary. All mice involved in the experiment were euthanized by inhalation of excessive isoflurane (5% concentration). We finally confirmed the occurrence of OP by analyzing the trabeculae of the distal femur using micro-CT.

### Treatment of the OVX mice

Ten 5-month-old OVX mice were randomly selected to receive treatments. An equal volume of PBS (100 μL), M2D-Exos^inhibitor-NC^ (100 μL, 10^10^ particles), or M2D-Exos^miR-486-5p inhibitor^ (100 μL, 10^10^ particles) was injected through the tail vein once a week. After two months of continuous injections, the femurs were dissected from the OVX mice to analyse differences.

### Micro-CT analysis

Prior to micro-CT scanning, mice femurs were immobilized with 4% paraformaldehyde at 4°C for 48 hours, and subsequently the formaldehyde was replaced with 75% ethanol. Images of femoral trabeculae were taken using a microCT scanner (Perkin Elmer, Waltham, MA, USA). The microCT analysis of femoral trabeculae was carried out based on previous studies [48].

### Histological analysis

#### Calcein staining

Mice were injected intraperitoneally with calcein (0.5 mg/each) (#S19167, Yuanye Bio-Technology Co, Shanghai, China). We gave the first injection on the 10th day before execution and the second injection on the 2nd day before execution. The paraformaldehyde-fixed femoral samples were cut into 5-μm sections, and the longitudinal sections of the samples were subsequently stained with calcium to detect bone formation. Results of femoral calcein staining are presented by fluorescence microscopy.

#### Tartrate-resistant acid phosphatase (TRAP) staining

Mice femurs were subjected to paraformaldehyde (4%) fixation and decalcification with EDTA (10%), and then the treated samples were dehydrated and paraffin-embedded. Femurs of each group were sectioned and stained for TRAP. Multinucleated TRAP-positive cells in the sections were visualized using a microscope (ZEISS, Axio Vert.A1, Germany).

#### Haematoxylin-eosin staining (H&E) and masson staining

The bone formation ability of mice was detected by H&E. After sacrifice, the femurs of the test mice were dissected. Mice femoral tissue was cut into 3 μm thick sections and stained with Masson and H&E at 30°C for 120 min. Histologic differences were analysed using an Olympus VS120 (Olympus, Japan).

### Human bone samples

Bone tissue samples were obtained from twelve female patients with OP at ages ranging from 55 to 70 years (T ≤ −2.5), as well as twelve female subjects without OP at ages ranging from 18 to 54 years (T > −2.5). The bone samples were obtained by the Department of Orthopaedics, Qilu Hospital of Shandong University. All patients were excluded from metabolic and endocrine diseases. This project was approved by the Medical Ethics Committee of Qilu Hospital of Shandong University (KYLL-2019-2-052).

### Statistical analysis

The data are presented as the mean ± SD, and SPSS 22.0 software was used for statistical analyses. Two independent groups were compared via Student’s *t* tests, and comparisons of multiple groups were made by one-way ANOVA. A *p* value < 0.05 indicated statistical significance.

### Availability of data and materials

All data generated and analysed during this study are included in this published article and supplementary information file.

## Supplementary Materials

Supplementary Figures
