# Supplementary material for: M2 macrophage-derived exosomal miR-486-5p influences the differentiation potential of bone marrow mesenchymal stem cells and osteoporosis
Source: Aging (Albany NY). 2023 Sep 25;15(18):9499–520. doi: 10.18632/aging.205031 (PMC10564417; doi:10.18632/aging.205031)
Supplement: Supplementary Figures [file aging-15-205031-s001.pdf]

SUPPLEMENTARY FIGURES

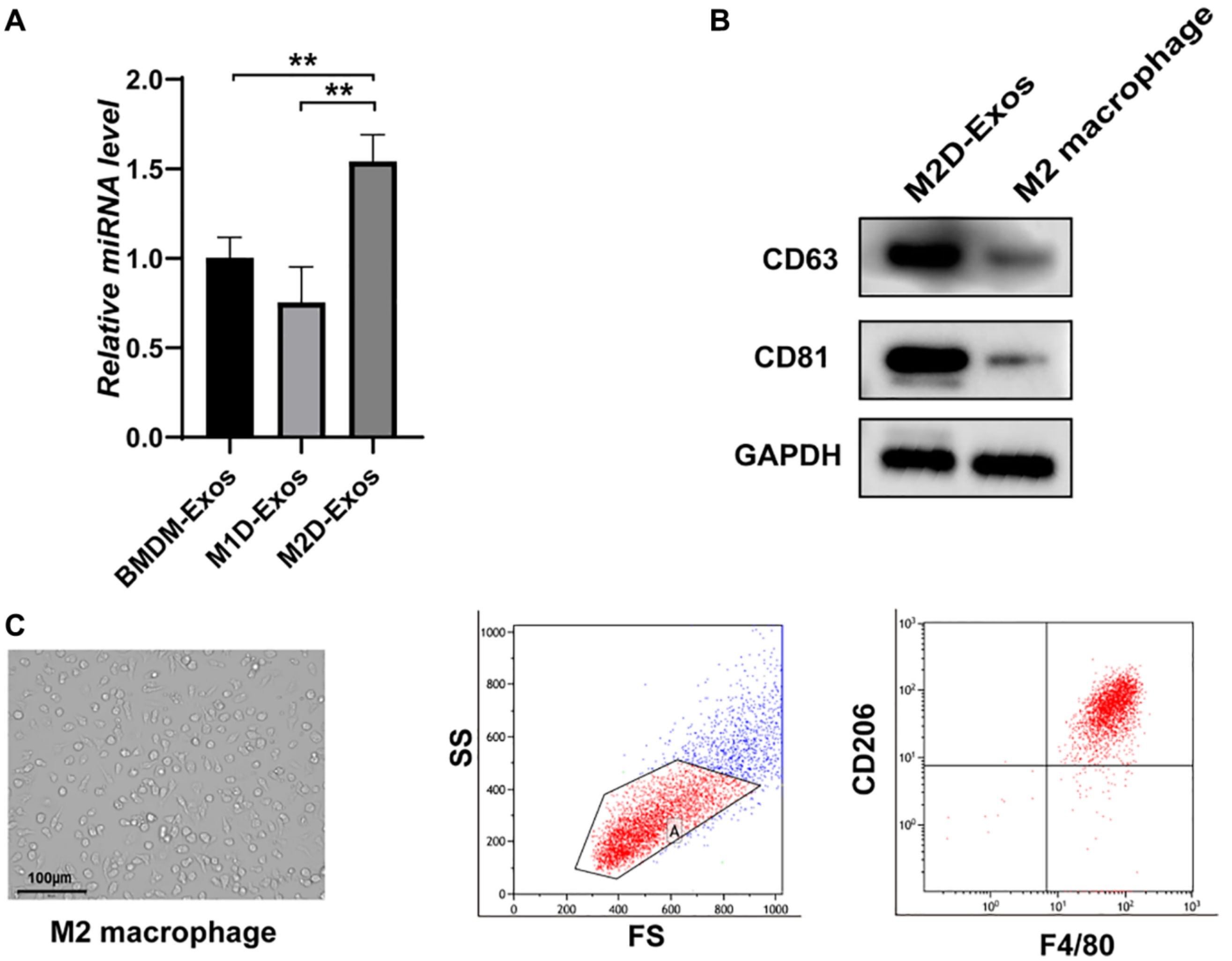

Supplementary Figure 1. (**A**) The miR-486-5p levels in monocyte-derived exosomes, MlD-Exos, and M2D-Exos were measured by qRT-PCR analysis. (**B**) The surface markers (CD63 and CD81) of exosomes were detected by western blotting. (**C**) M2 macrophages were identified by flow cytometry. Data are expressed as the mean ± SEM, $^{*}p < 0.05$, $^{**}p < 0.01$, $^{***}p < 0.005$.

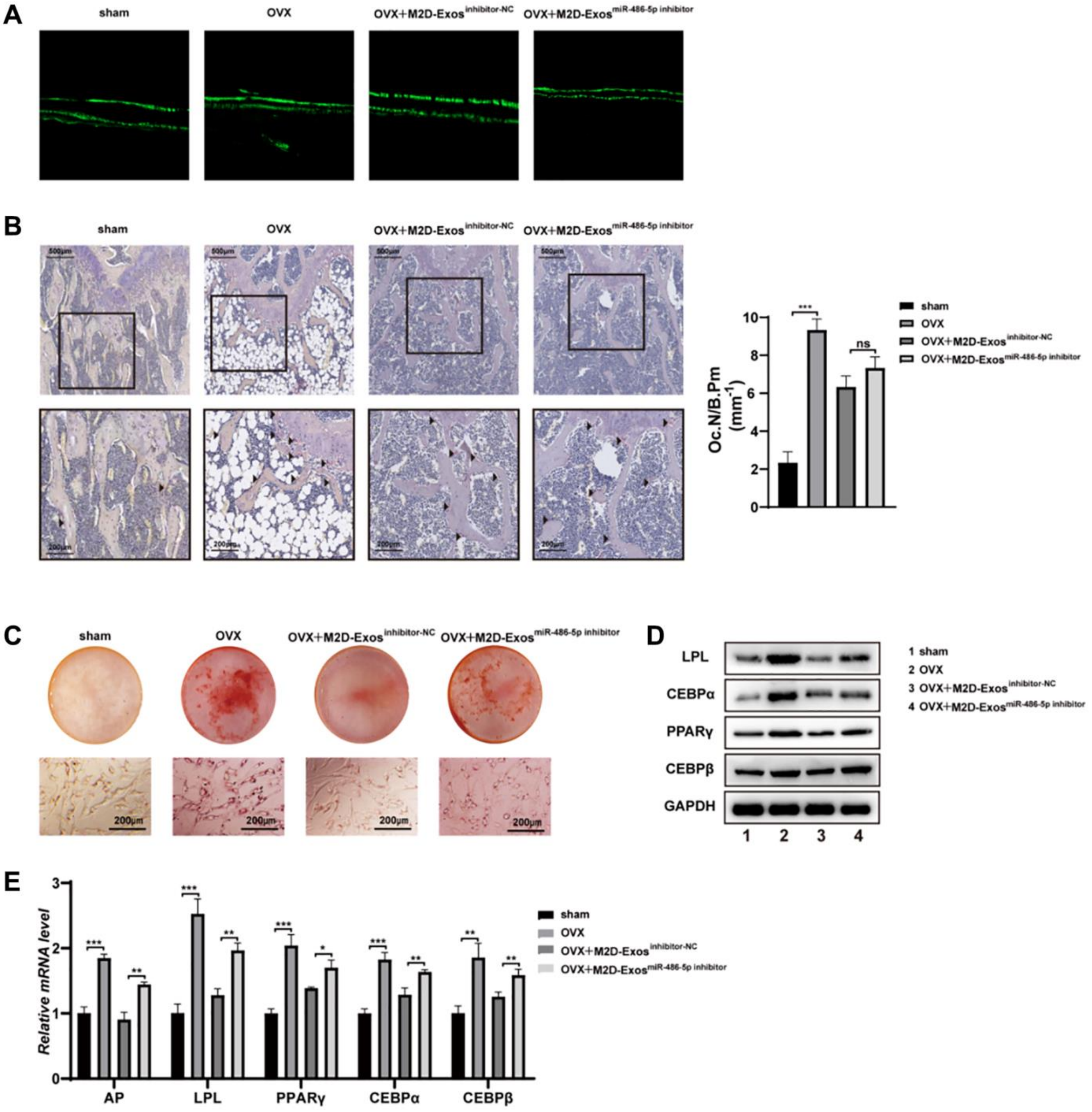

Supplementary Figure 2. (**A**) Representative images of new bone formation assessed by double calcein labelling in the femur sections. (**B**) Representative images show tartrate-resistant acid phosphatase (TRAP) staining and osteoclast number per bone perimeter (Oc.N/B.Pm) in the femur sections of different groups. (**C**) Oil red 0 staining was performed to detect lipid droplet formation in different groups in BMMSCs. (**D**, **E**) Western blotting and qRT-PCR were performed to analyse the protein and mRNA expression levels of adipogenic markers, respectively. Data are expressed as the mean ± SEM, $^{*}p < 0.05$, $^{**}p < 0.01$, $^{***}p < 0.005$.

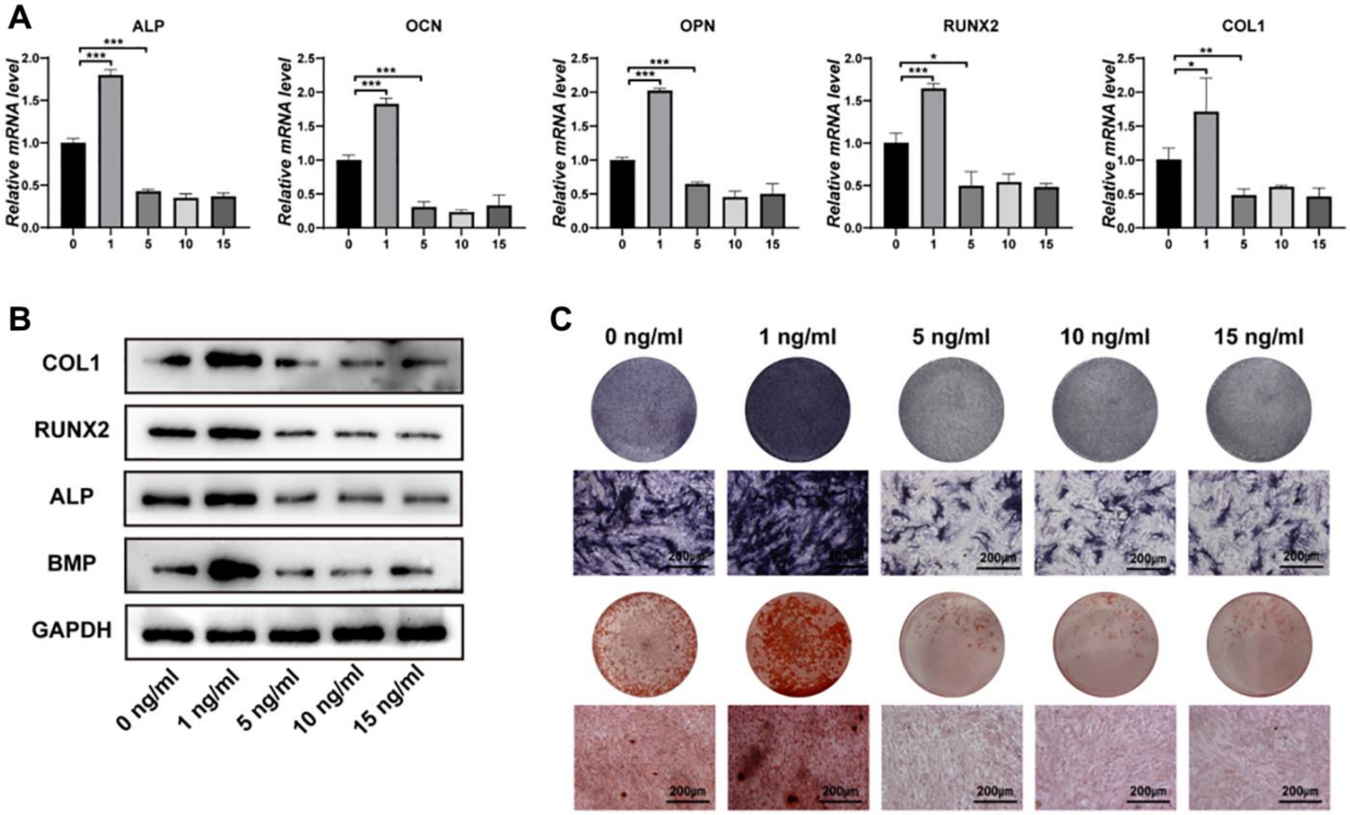

Supplementary Figure 3. The effect ofTGF-β 1 on osteogenic differentiation in BMMSCs. (**A**, **B**) Osteoblast marker genes were detected by qRT-PCR and western blot after different concentrations of TGF-β I treatment. (**C**) ALP staining and ARS were performed after different concentrations of TGF-β I treatment. Data are expressed as the mean ± SEM, $^{*}p < 0.05$, $^{**}p < 0.01$, $^{***}p < 0.005$.
